# The incidence of infusion reactions associated with monoclonal antibody drugs targeting the epidermal growth factor receptor in metastatic colorectal cancer patients: A systematic literature review and meta‐analysis of patient and study characteristics

**DOI:** 10.1002/cam4.2413

**Published:** 2019-08-03

**Authors:** Lauren C. Bylsma, Rebecca Dean, Kimberly Lowe, Laura Sangaré, Dominik D. Alexander, Jon P. Fryzek

**Affiliations:** ^1^ EpidStat Institute Ann Arbor Michigan USA; ^2^ Amgen One Amgen Center Drive Thousand Oaks California USA; ^3^ SimulStat Portland Oregon USA; ^4^ EpidStat Institute Rockville Maryland USA

**Keywords:** anti‐EGFR monoclonal antibody, infusion reaction, meta‐analysis, metastatic colorectal cancer, systematic review

## Abstract

**Background:**

Systemic cancer therapies may induce infusion reactions (IRs) or hypersensitivities. Metastatic colorectal cancer (mCRC) patients treated with anti‐EGFR therapies, including cetuximab and panitumumab, may be subject to these reactions. We conducted a meta‐analysis to estimate the IR incidence in this population and identify variations in this incidence by patient or study characteristics.

**Methods:**

A systematic review was conducted to identify observational studies or clinical trials of mCRC patients treated with anti‐EGFR therapies that reported occurrences of IRs, hypersensitivity, or allergy/anaphylaxis. The objective of the study was to estimate the incidence of IRs. Random effects models were used to meta‐analyze the incidence of IRs overall and stratified by therapy type, study design, geographic location, *RAS or KRAS* mutation status, grade of reaction severity, and terminology used to describe the reaction.

**Results:**

The pooled estimate for IR incidence was 4.9% (95% confidence interval: 3.6%‐6.5%). Lower‐grade reactions were more common than higher‐grade reactions overall and the incidence of reactions among cetuximab patients was nearly four times that of panitumumab patients (6.1% vs 1.6%).

**Conclusions:**

IRs occur in approximately 5% of mCRC patients treated with anti‐EGFR therapies, and the incidence varies significantly by grade of severity and therapy type. Studies evaluating these outcomes should consider investigating survival outcomes by IR status to determine its prognostic relevance.

## Graphical abstract

Infusion reactions and hypersensitivities have been reported among metastatic colorectal cancer patients treated with anti‐EGFR therapies, but the summary incidence has not previously been characterized. This systematic review and meta‐analysis demonstrated that the summary incidence of infusion reactions was 4.9% in this population.

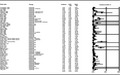

## 1 INTRODUCTION

Colorectal cancer (CRC) is the third most commonly diagnosed cancer worldwide, and the public health burden of treating this malignancy is well‐recognized. Approximately, 20%‐25% of new cases of colorectal cancer are metastatic (mCRC) at diagnosis and up to 50% of all patients eventually develop metastatic disease.^1^, ^2^, ^3^, ^4^ During the last decade, improvements in the treatment of mCRC patients have increased the median survival from 12 to 21 months.^5^ Survival improvements can be attributed to the development of the antiangiogenic agent bevacizumab and monoclonal antibody (mAb) therapies that target the epidermal growth factor receptor (EGFR), primarily panitumumab and cetuximab.^6^

Most systemic cancer treatments have the potential to cause infusion reactions (IRs).^7^, ^8^, ^9^ These reactions can either be IgE‐mediated (allergic, type 1 hypersensitivity) or non‐IgE‐mediated (anaphylactoid, nonallergic) and can vary in severity.^8^ Although the exact mechanism for IR in mAb therapy is unknown, possible mechanisms include cytokine‐release syndrome, type 1 hypersensitivity, IgG anaphylaxis, complement activation, or degranulation of mast cells and basophils.^10^ Reactions to platinum‐based therapies are adaptive and tend to occur after six to eight infusions, while reactions to taxanes and mAbs generally occur on the first or second infusion.^11^ Anaphylactic reactions tend to occur within minutes of the infusion and increase in severity, while symptoms of cytokine‐release syndrome may occur within 30‐120 minutes of beginning the infusion.^8^ Early signs of an IR include changes in respiratory, cardiac, or integumentary status.^12^ Other clinical manifestations can include dizziness, confusion, anxiety, arthralgias, fever, nausea, and vomiting.^8^ Severe reactions typically result in rapid onset of airway obstruction. Asthmatic or atopic patients may be at an increased risk of developing hypersensitivity to therapy infusions, and other risk factors include concurrent autoimmune disease, iodine or seafood allergies, preexisting cardiac or pulmonary dysfunction, and taking higher than standard drug doses.^8^

IR in response to anti‐EGFR mAb drugs generally occur far less frequently than other therapies, although the reported range of incidence is wide (0%‐33%).^13^ These proportions have been reported to vary by drug, as the incidence of IR associated with cetuximab ranged from 7.6%‐33%, whereas the proportions associated with panitumumab ranged from 0%‐4%.^13^ The cetuximab label reports IR incidence as occurring in approximately 3% of patients,^14^ and the panitumumab label reports an incidence of approximately 1%.^15^

Research is needed to identify patients who are at risk of IRs during treatment with anti‐EGFR therapies. The global incidence of IRs has yet to be summarized for mAbs against EGFR in mCRC patients by study characteristics, including geographic location, study design (observational versus clinical trial), gender, and other reported subgroups. Thus, using information obtained from the published literature, this systematic literature review provides a comprehensive, worldwide assessment of the incidence of IR among mCRC patients taking anti‐EGFR mAb therapies by study and patient characteristics.

## 2 MATERIALS AND METHODS

This systematic literature review and meta‐analysis followed the 2015 Preferred Reporting Items for Systematic Review and Meta‐Analysis Protocols (PRISMA‐P).^16^

### 2.1 Literature search and eligibility criteria

PubMed, Embase, Web of Science, https://ClinicalTrials.gov, and The Cochrane Library databases were queried for relevant peer‐reviewed studies using the following search string (with synonyms and closely related terms): “colorectal”, “neoplasms”, “metastatic”, “EGFR”, “infusion reaction”, “hypersensitivity” and “incidence”. The search strategy was adapted to meet the search specifications of each included database. Observational studies, randomized controlled trials, or nonrandomized trials reporting proportion of patients experiencing IR in adult mCRC patients treated with anti‐EGFR mAb therapies published between 1 January 2000 and 16 December 2017 were included. As cetuximab was approved in the US in 2004 and panitumumab in 2006, studies published prior to 2000 were unlikely to capture any relevant trials or observational studies.^14^, ^15^ Studies reporting the incidence of hypersensitivity reactions, allergic reactions, and anaphylaxis were also included. Case reports, editorials, letters to the editor, comments, and practice guidelines were excluded as were studies not published in English and studies conducted in pediatric or animal populations.

### 2.2 Study selection

The same search string was run in multiple databases, resulting in some duplicate studies that were removed. After de‐duplication across databases, the remaining articles were screened at the levels of abstract and full text by at least two reviewers. Where multiple publications reported on the same cohort or trial, one reviewer evaluated all publications and selected the most updated and inclusive publication from each cohort/trial to avoid duplication. In addition, the reference sections of relevant review publications were screened to identify any additional articles that provided pertinent data but were not captured in our electronic literature searches.

### 2.3 Data collection process

We abstracted information on study design characteristics, population demographics, treatment data, outcomes, sex, age, race, geographic location, years of study data collection, length of follow‐up, sample size, patient comorbidities, location of the primary tumor, stage at diagnosis, and type of therapy. The incidence of IR, hypersensitivity, and/or allergy/anaphylaxis was abstracted as the outcome of interest. As a measure of quality control, each abstraction was assessed for accuracy by at least two independent reviewers.

### 2.4 Risk of bias in individual studies

The Cochrane Risk of Bias Assessment Tool was used to evaluate the risk of bias in individual studies.^17^ The tool includes seven measures to evaluate the risk of selection bias, performance bias, detection bias, attrition bias, reporting bias, or other forms of bias. By conducting in‐depth review of these study parameters, we were able to determine if there were qualitative differences within the studies that would prevent the combining of quantitative data across the studies. In addition, these measures of internal and external validity provided a more comprehensive scientific foundation to interpret the quantitative evidence.

### 2.5 Quantitative data synthesis

In addition to the overall analysis of IR incidence, subgroup analyses were conducted for several characteristics of interest, including geographic location, study design, therapy, Grade, *KRAS* status, dates study was conducted, median age, category of study size, and percent male in cohort. Several studies included both a measure of overall IR incidence as well as incidence of hypersensitivity and/or allergy/anaphylaxis. In these cases, only the aggregate measure of IR incidence was included in the overall analyses in order to prevent weighing the study twice. However, additional measures were included in their respective categories in the “Description of reaction” subgroup analyses. Similarly, for the analysis of IR by grade, if a study reported the incidence of reactions by severity grade, each estimate was included in the respective subgroup, whereas the estimate of all IR regardless of grade was used in the overall analysis. Multiple studies only reported grades 3/4 adverse events. Although these estimates were included in the overall analysis, the stratification by grade allows for an evaluation of heterogeneity in the variation of incidence by Grade of severity. If a study provided stratified estimates by *KRAS* status, each estimate was included in the respective *KRAS* subgroup, whereas multiple studies were conducted in populations of solely *KRAS* wild‐type patients and thus were only included in the *KRAS* wild‐type subgroup.

### 2.6 Analysis

A narrative synthesis of the studies included in the review was first performed. Meta‐analyses for the overall incidence of IRs as well as for subgroups were performed using random effects models, which accounts for both within‐ and between‐study variability. Studies were weighted by the inverse of their variance per the method proposed by DerSimonian and Laird.^18^ Statistical heterogeneity across studies for the overall and subgroup analyses was assessed using the Cochran's Q test and *I* ^2^ statistic, which indicate the percentage of variation attributable to between‐study heterogeneity.^19^ Forest plots were produced to illustrate the individual and summarized incidence estimates and 95% confidence interval (CI).

Although the original analysis plan included a meta‐analysis of overall and progression‐free survival by IR status (yes vs no), none of the studies included in the meta‐analysis reported survival in this manner. Therefore, we conducted a hypothesis‐generating exercise by comparing overall and progression‐free survival in studies reporting high incidences of IR (≥75th percentile of reaction incidence) with studies reporting low incidences of IR (<75th percentile). An observed trend in survival between these groups may encourage research evaluating survival by IR status in future studies.

### 2.7 Risk of bias across studies

Publication bias in the overall meta‐analysis was assessed using the methods described by Sterne and Egger and visually using a funnel plot of precision by logit event rate.^20^ Egger's regression was used to test for the presence of publication bias. If the presence of publication bias was identified, an analysis using the trim and fill method as described by Duval and Tweedie^21^ was planned to evaluate the adjusted IR incidence estimate adding in hypothetical “missing” (unpublished) studies, acknowledging that this is a theoretical exercise. Comprehensive Meta‐Analysis, Version 3.3.070 was used to conduct the analyses.

## 3 RESULTS

### 3.1 Study selection

**Figure 1:**
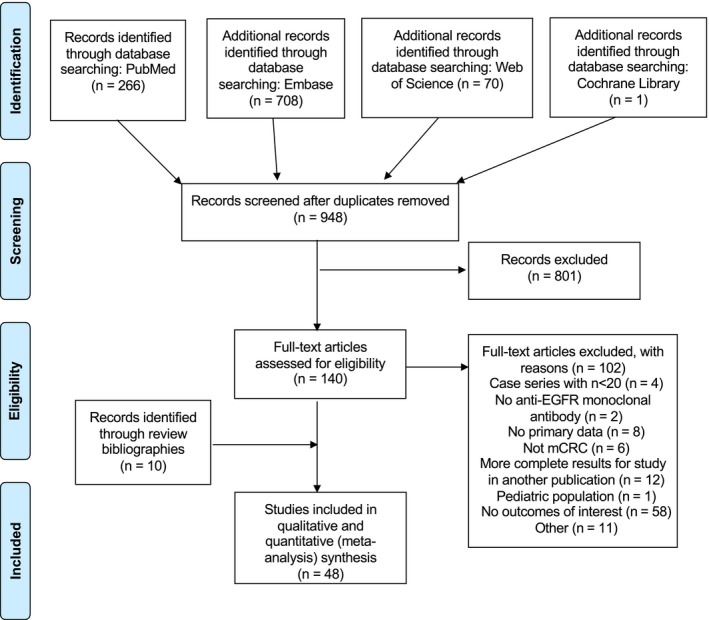
PRISMA flow diagram of study inclusion

The process of study selection is demonstrated in the PRISMA flow diagram of study inclusion (Figure 1). Briefly, 948 unique publications were identified from PubMed, Embase, Web of Science, and the Cochrane Library. Abstract review by two independent reviewers (LB and RD) resulted in full‐text assessment of 140 publications for relevant information. Ten additional publications were identified through the review of bibliographies of these articles. After final assessment, 48 articles (12 903 patients total) were included in the meta‐analysis.

### 3.2 Study characteristics

Study and patient characteristics for the 48 studies included in the meta‐analysis are demonstrated in Table S1. Most of the studies were randomized or nonrandomized clinical trials (n = 35; 73%) or observational studies (n = 12; 25%). One study (O'Neil 2007^22^) included retrospective results from both clinical trials and observational studies at two centers. Eighteen (37.5%) studies were conducted in Europe, 11 (23%) in the United States, 7 (15%) in Japan, and 12 (25%) in other counties or multiple areas. Study sizes ranged from 21 patients (Folprecht 2006^23^) to 2006 patients (Yamaguchi 2014^24^). Only one study included more female patients (51.5%) than male (Emons 2011^25^). The median age of patients in the studies ranged from 54.6 years old (Tang 2017^26^) to 79 years old (Kienle 2015^27^). Most studies evaluated cetuximab (n = 36; 75%) in at least one of their arms while 15 studies (31%) included panitumumab in one of their arms. Nineteen studies (40%) used pretreatment medication, typically antihistamines and/or corticosteroids, before administration of the infusion.

### 3.3 Risk of bias

**Figure 2:**
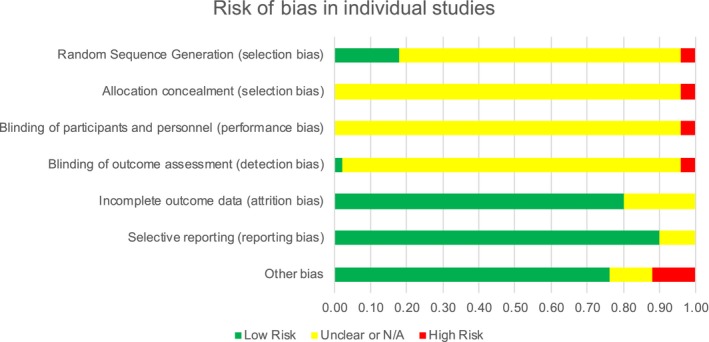
Risk of bias in individual studies

The risk of bias in individual studies is presented in a summary graph in Figure 2. A low risk of bias was observed in most studies in terms of attrition, reporting, and other biases. Selection, performance, and detection biases were either not described in many studies or were not applicable as in the cases of open‐label or observational studies, resulting in a large proportion of “unclear or not applicable.” Several studies did not report funding sources or conflicts of interest, resulting in a high risk of “other” bias.

### 3.4 Meta‐analysis of infusion reaction incidence

**Figure 3:**
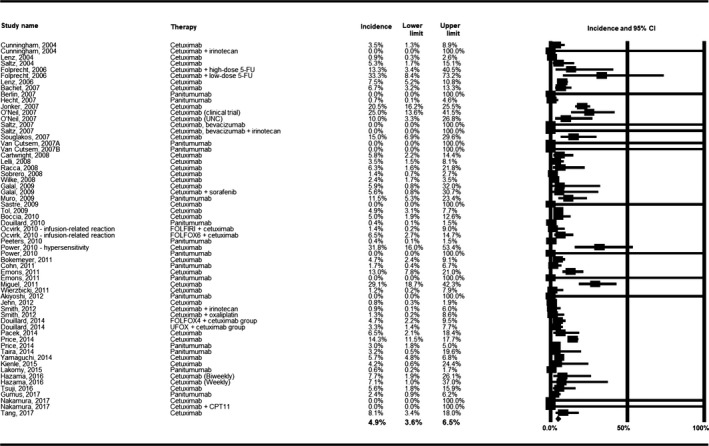
Meta‐analysis of the incidence of infusion reaction

The IR incidence summary estimate in studies of mCRC patients treated with anti‐EGFR mAB therapies was 4.9% (95% CI: 3.6%‐6.5%) (Table 1, Figure 3). Statistical heterogeneity was present in this analysis (*P*‐value for heterogeneity [*P*‐Het]: <0.001; *I* ^2^ value: 85.3%). The results of sensitivity analyses by selected study characteristics are shown in Table 1 The incidence of IR in patients treated with cetuximab was nearly four times that of patients treated with panitumumab (6.1% vs 1.6%; *P* = 0.001). All studies reporting that patients received pretreatment before infusion were conducted in patients receiving cetuximab and all studies reporting that patients were not pretreated before infusion were conducted in patients given panitumumab; thus the results of this subgroup analysis were not more informative than the subgroup analysis by therapy type.

**Table 1:** Meta‐analysis of the incidence of infusion reactions by selected study characteristics

Analysis	N*	Incidence estimate (95% CI)	*P*‐Het; I^2^	*P*‐value*
Overall	61	4.9% (3.6%‐6.5%)	<0.001; 85.3%	
Therapy				
Panitumumab	15	1.6% (0.8%‐3.3%)	<0.001; 64.3%	**0.001**
Cetuximab	46	6.1% (4.6%‐8.2%)	<0.001; 85.7%	
Description of reaction^a^				
Infusion reaction	24	4.6% (2.9%‐7.2%)	<0.001; 88.0%	**0.024**
Infusion‐related reaction	14	2.6% (1.7%‐4.1%)	0.011; 52.6%	
Hypersensitivity	16	8.0% (4.4%‐14.2%)	<0.001; 82.1%	
Allergic reaction/anaphylaxis	15	3.1% (1.7%‐5.7%)	<0.001; 73.1%	
Grade^a^				
≤2	12	8.9% (5.5%‐14.0%)	<0.001; 82.5%	**<0.001**
≥3	37	2.8% (1.9%‐4.0%)	<0.001; 76.9%	
All Grades Combined/Grade Not Specified	26	6.2% (4.0%‐9.3%)	<0.001; 82.9%	
Study design				
Clinical trial	45	4.9% (3.5%‐6.9%)	<0.001; 81.7%	0.891
Observational	16	4.7% (2.7%‐8.1%)	<0.001; 89.6%	
*KRAS* status^a^				
Wild‐type	13	4.2% (2.1%‐8.3%)	<0.001; 85.8%	0.570
Mutant	4	6.1% (2.0%‐17.4%)	0.862; 0.0%	
Dates study was conducted				
Pre‐2008	26	5.2% (2.9%‐9.1%)	<0.001; 88.7%	0.781
Includes 2008	16	4.1% (2.4%‐6.8%)	<0.001; 84.5%	
Post‐2008	14	5.1% (2.7%‐9.3%)	<0.001; 73.4%	
Percent male in cohort				
≤63%	27	4.4% (3.2%‐5.8%)	<0.001; 66.9%	0.416
>63%	34	5.5% (3.4%‐8.7%)	<0.001; 88.3%	
Median age in cohort				
≤60	20	5.7% (3.7%‐8.8%)	<0.001; 64.8%	0.438
>60	41	4.6% (3.2%‐6.5%)	<0.001; 88.7%	
Study location				
Europe	22	4.4% (2.7%‐7.2%)	<0.001; 83.2%	0.227 (large group^b^); 0.345 (small group^c^)
Southern Europe	5	10.8% (3.6%‐28.1%)	<0.001; 81.9%	
Western Europe	8	4.2% (1.8%‐9.2%)	<0.001; 77.1%	
Central/Eastern Europe	4	2.6% (0.7%‐9.1%)	0.001; 80.5%	
North America (USA and Canada)	15	7.1% (3.7%‐13.1%)	<0.001; 67.8%	
USA	14	8.1% (4.3%‐14.7%)	<0.001; 66.0%	
Asia	12	5.9% (5.0%‐7.0%)	0.966; 0.0%	
Japan	9	5.9% (5.0%‐7.0%)	0.891; 0.0%	
Multi‐country	12	3.1% (1.5%‐6.4%)	<0.001; 94.0%	
Study sample size				
<50	22	13.7% (10.0%‐18.4%)	0.418; 3.2%	**<0.001**
50‐100	11	6.1% (3.3%‐11.0%)	<0.001; 75.2%	
100‐200	13	3.9% (2.5%‐6.0%)	0.006; 56.9%	
>200	15	2.7% (1.5%‐4.7%)	<0.001; 95.1%	

Abbreviations: *P*‐Het: *P*‐value for heterogeneity.

a If multiple estimates from a study were provided for different subgroup categories, each estimate was included in its respective category.

b Large Group: Europe, North America, Asia, Multi‐country.

c Small Group: Southern Europe, Western Europe, Central/Eastern Europe, USA, Japan.

* N refers to the number of data points from unique treatment arms/cohorts included out of 47 studies.

The bolded p‐values indicate *p* < 0.05.

The incidence of IR varied significantly by the terminology used to describe the reaction (*P* = 0.024): studies reporting “hypersensitivity” to infusions had the highest incidence of reactions (8.0%) and studies reporting “infusion‐related reactions” had the lowest incidence (2.6%). Additionally, IR incidence varied by grade of reaction. The incidence of Grade ≤ 2 reactions was 8.9%, while the incidence of Grade ≥ 3 reactions was 2.8% and the incidence of all grades combined/grade not specified was 6.2% (*P* < 0.001). A significant difference was also observed by approximate quartiles of sample size, with the highest incidence of reactions (13.7%) among studies with < 50 patients and the lowest incidence (2.7%) among studies with > 200 patients. A univariate meta‐regression was run on study size as a continuous variable and demonstrated a significant decrease of IR incidence with increase in study sample size. No statistically significant differences were observed within the subgroup categories of study design (clinical trial vs observational), KRAS status (wild‐type vs mutant), dates study was conducted (pre‐ vs including vs post‐2008), percent male in cohort (≤63% vs >63%), median age in cohort (≤60 vs >60), or study location.

### 3.5 Publication bias

**Figure 4:**
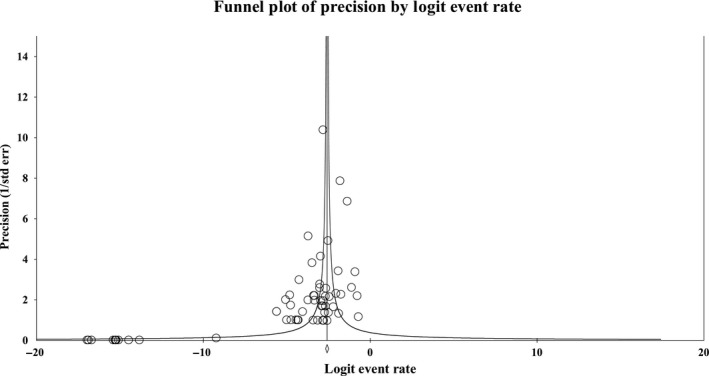
Funnel plot of precision by logit event rate

A visual assessment of the funnel plot for the overall incidence of IR (Figure 4) demonstrated some evidence of publication bias, as there were more studies on the left side of the mean logit event rate than the right. Egger's regression test confirmed the presence of possible publication bias (two‐tailed *P* = 0.044). Using Duval and Tweedie's method adding in “missing” studies to the right of the mean, the adjusted point estimate only differed from the original estimate in the lower confidence level estimate (adjusted: 4.9%; 95% CI: 3.7%‐6.5%), indicating the statistical robustness of the original estimate.

### 3.6 Survival analyses

The results of the exploratory survival meta‐analyses by higher vs lower incidence of IR are shown in Table 2. The overall and progression‐free survival among studies with IR incidences < 75th percentile were not significantly different than those with incidences ≥ 75th percentile. However, the studies with grade 3/4 IRs ≥ 75th percentile had significantly higher overall (19 vs 12.5 months) and progression‐free survival (8.3 vs 4.6 months) than studies with grade 3/4 IRs < 75th percentile.

**Table 2:** Meta‐analysis for survival of mCRC patients by the proportion of patients with an infusion reaction

	Overall survival estimates			Progression‐free survival estimates		
Characteristic	N	Median months (95% CI)	*P*‐Het; *I* ^2^	N	Median months (95% CI)	*P*‐Het; *I* ^2^
Proportion of cohort with infusion reaction						
<75th percentile	21	12.2 (11.9‐12.6)	*P* < 0.001; 95.6%	27	5.3 (3.9‐7.3)	*P* < 0.001; 99.3%
≥75th percentile	8	14.0 (9.2‐21.3)	*P* < 0.001; 95.4%	9	4.76 (2.9‐7.8)	*P* < 0.001; 94.1%
Proportion of cohort with grade 3‐4 infusion reactions						
<75th percentile	16	12.5 (10.7‐14.7)	*P* < 0.001; 94.7%	20	4.6 (3.2‐6.7)	*P* < 0.001; 99.5%
≥75th percentile	6	19.0 (17.7‐20.3)	*P* = 0.770; 0.0%	7	8.3 (7.5‐9.2)	*P* < 0.001; 95.3%
Not reported	7	11.4 (7.3‐17.8)	*P* < 0.001; 94.1%	9	4.8 (3.2‐7.1)	*P* < 0.001; 90.6%

## 4 DISCUSSION

This systematic review and meta‐analysis summarized the available data on IRs in mCRC patients treated with anti‐EGFR mAbs, resulting in an overall incidence estimate of nearly 5%. Statistically significant heterogeneity was present in this analysis. Subgroup analyses were conducted to evaluate potential sources of heterogeneity and identified significant variation by therapy type, pretreatment status, reaction description, study sample size, and grade of reaction.

The results of this study updated and expanded upon the 2012 systematic literature review by Song et al^13^ Although narrative in nature, their review reported incidences for cetuximab ranging from 7.6%‐33% in clinical trials and 27%‐32% in observational studies. We observed IR incidences for cetuximab ranging from 0%‐33% in clinical trials and 0.65%‐32% in observational studies. For panitumumab studies, Song et al reported incidence proportions ranging from 0%‐0.7% among clinical trials and did not identify any observational studies reporting IR. In the current study, IR incidence reported among clinical trials ranged from 0%‐12% and observational studies ranged from 0%‐2.2%.

Egger's regression test demonstrated statistically significant publication bias in the overall analysis as several studies reported no events of IRs. It would be expected that if a study observed a very high incidence of IRs that it would be reported. Indeed, we observed that studies with smaller sample sizes reported larger incidences of IRs than studies with larger sample sizes in both categorical and continuous analyses. This may suggest the presence of the “small study effect” in this analysis, a phenomenon where smaller studies show larger treatment effects than larger studies.^28^ Due to the summary estimate of IR incidence being approximately 5% across all studies, it would be expected that most of the smaller studies would report no events observed. Of the studies with a sample size < 50 patients (n = 22 treatment arms), seven reported no IR events. We hypothesize that there may be some publication bias present among the smaller studies where either those with no IR events are not published, or published smaller studies do not report a 0% incidence of IR.

Subgroup analyses also identified a higher incidence of lower‐grade IR (grade ≤ 2) than higher‐grade IR (grade ≥ 3), and a significant difference between terminology used to describe reactions. Although the National Institutes of Health have published within the Common Terminology Criteria for Adverse Events a grading scale for severity infusion‐related reactions,^29^ this scale may not be used globally and often grading criteria used to define IR severity in published studies are unclear.^8^ The terminology used to describe IR, for example, “hypersensitivity,” “allergic reaction,” or “infusion‐related reaction,” also varies by package insert.^8^ In this summary, we were limited by how study investigators characterized and defined IRs. Improved standardization of terminology and grading would aid both clinicians and researchers in the diagnosis and evaluation of IRs.

The strength of this study includes the large body of scientific evidence available to meta‐analyze the incidence of IRs in anti‐EGFR mAb‐treated mCRC patients and identify significant variations in incidence by several factors. Multiple databases were searched to identify publications and nearly 13 000 patients were included in these analyses. There may be some publication bias present in these analyses, particularly among the studies with small sample sizes, and the meta‐analysis of survival outcomes by IR status was limited by the currently published data. Despite these limitations, the results of these meta‐analyses indicate that IRs, although a relatively uncommon event, present a unique challenge for providers and patients undergoing therapy with anti‐EGFR mAbs.

## 5 CONCLUSION

This systematic review and meta‐analysis reported a summary incidence estimate for IRs of 5% among mCRC patients treated with anti‐EGFR mAbs. IR incidence was higher among patients treated with cetuximab than panitumumab and was lower among studies with larger sample sizes than those with smaller sample sizes. Grades 1 and 2 reactions were more common than higher‐grade reactions. An evaluation of survival by IR status is recommended for future trials and observational studies to determine the prognostic value of IRs in this population.

## CONFLICT OF INTEREST

LCB, DDA, and JF are employees of EpidStat Institute, which received funding for this work. EpidStat has also received funding from Amgen, Inc, AstraZeneca, Genentech, Merck, and Sanofi for other research. RD consults for EpidStat Institute. KL is an employee of Amgen, Inc and owns stock in Amgen, Inc LS is employed by SimulStat, work contracted through Amgen, Inc

## AUTHOR CONTRIBUTIONS

Lauren C. Bylsma, methodology, data curation, investigation, formal analysis, visualization, writing‐original draft, and writing‐review and editing. Rebecca G. K. Dean, data curation, investigation, and writing‐review and editing. Kimberly Lowe, conceptualization, supervision, visualization, and writing‐review and editing. Laura Sangaré, visualization, and writing‐review and editing; Dominik D. Alexander, methodology, supervision, investigation, and writing‐review and editing. Jon Fryzek, conceptualization, supervision, methodology, visualization, writing‐original draft, and writing‐review and editing.
