# Supplementary material for: The incidence of infusion reactions associated with monoclonal antibody drugs targeting the epidermal growth factor receptor in metastatic colorectal cancer patients: A systematic literature review and meta‐analysis of patient and study characteristics
Source: Cancer Med. 2019 Aug 3;8(12):5800–9. doi: 10.1002/cam4.2413 (PMC6745824; doi:10.1002/cam4.2413)
Supplement: Supplementary file 1 [file CAM4-8-5800-s001.docx]

**Supplemental Table 1. Descriptive characteristics of included studies*.**

| **Author, Year** | **Study Design** | **NCT number and name of trial/cohort** | **Study country** | **N** | **% male** | **Median Age** | **Treatment regimen** | **Use of Pre-treatment medication** | **Description of Infusion Reaction** |
| --- | --- | --- | --- | --- | --- | --- | --- | --- | --- |
| Akiyoshi, 2012^1^ | Prospective multicenter Phase II study | NR “SHIP” | Japan | 48 | 56.3% | 62 | Panitumumab +/- irinotecan: 89.58% | NR | Infusion reaction |
| Bachet, 2007^2^ | Retrospective observational | NR | France | 105 | 59.1% | 60.3 | Cetuximab either alone or with irinotecan, FOLFIRI, or chronomodulated FOLFIRI | NR | Allergic reaction/anaphylaxis |
| Berlin, 2007^3^ | Phase II study | NR | United States | 43 | Part 1: 84.2% Part 2: 58.3% | 58.65 | Panitumumab + IFL (Part 1), then Panitumumab + FOLFIRI (Part 2) | NR | Infusion-related reaction |
| Boccia, 2010^4^ | Phase II trial | NR | United States | 82 | 51.2% | 61 | Cetuximab + FOLFOX6 | Antihistamine | Hypersensitivity |
| Bokemeyer, 2011^5^ | Retrospective analysis of a Phase II trial | NCT00125034 “OPUS” | Europe | 337 | 53.7% | 62 | Cetuximab + FOLFOX-4 + | NR | Infusion-related reaction |
| Cartwright, 2008^6^ | Phase II, open-label, non-randomized trial | NR | United States | 69 | 61.0% | 61.5 | Cetuximab + XELIRI | NR | Allergic reaction/anaphylaxis |
| Cohn, 2011^7^ | Phase II trial | NCT00411450 “PRECEPT” | United States | 115 | 67.0% | 60.2 | Panitumumab + FOLFIRI | NR | Infusion reaction |
| Cunningham, 2004^8^ | Open-label randomized trial | NR | Multi-country (Europe) | 218 | 65.6% | 59 | Cetuximab + irinotecan | Antihistamine | Hypersensitivity |
|  |  |  |  | 111 | 56.8% | 58 | Cetuximab |  |  |
| Douillard, 2010^9^ | Phase III trial | NCT00364013 “PRIME” | Multi-country | 1096 | 63.3% | 62.5 | Panitumumab + FOLFOX-4 | Only after infusion reaction occurred (unspecified type) | Infusion reaction |
| Douillard, 2014^10^ | Multicenter, randomized, open-label, phase II study | NCT00439517 “FUTURE” | Multi-country | 150 | 63.0% | 61.5 | Cetuximab + FOLFOX4 | NR | Infusion-related reaction |
|  |  |  |  | 132 | 63.0% | 60 | Cetuximab +  UFOX |  |  |
| Emons, 2011^11^ | Retrospective chart review | NR | United States | 103 | 48.5% | 57.6 | Bevacizumab, cetuximab, or panitumumab | NR | Infusion reaction |
| Folprecht, 2006^12^ | Open-label, uncontrolled phase I/II trial | NR | Germany | 6 | 67.0% | 61.5 | Cetuximab + Low-dose 5-FU | Antihistamine | Hypersensitivity |
|  |  |  |  | 15 | 80.0% | 62 | Cetuximab + High-dose 5-FU |  |  |
| Galal, 2009^13^ | Phase II trial | NR | Saudi Arabia | 17 | 64.7% | 58 | Cetuximab | Antihistamine | Infusion reaction |
|  |  |  |  | 18 | 61.1% | 57 | Cetuximab + sorafenib |  |  |
| Gumus, 2017^14^ | Retrospective observational | NR | NR | 168 | 64.0% | 60 | Panitumumab | NR | Infusion reaction |
| Hazama, 2016^15^ | Phase II trial | UMIN000007195 “FLEET2” | Japan | 14 | 64.3% | 71.5 | Weekly cetuximab + XELOX | Serotonin receptor antagonists, corticosteroids, antihistamines, and selective neurokinin 1 receptor antagonists | Infusion reaction |
|  |  |  |  | 26 | 61.5% | 65.5 | Biweekly cetuximab + XELOX |  |  |
| Hecht, 2007^16^ | Phase II, open-label, multicenter study | NR | United States | 148 | 56.1% | 59.5 | Panitumumab | Premedication was not required. | Infusion reaction |
| Jehn, 2012^17^ | Non-interventional, uncontrolled multi-center study | NR | Germany | 614 | 65.0% | 65 | Cetuximab +/- chemotherapy (irinotecan, 5-fluorouracil/ folinic acid, oxaliplatin, capecitabine, gemcitabine and mitomycin C) | NR | Allergic reaction/anaphylaxis |
| Jonker, 2007^18^ | Open-label, randomized controlled trial | NCT00079066 | Canada and Australia | 287 | 64.8% | 63 | Cetuximab + BSC | Antihistamine | Infusion reaction |
| Kienle, 2015^19^ | Phase II trial | NR “SAKK 41/10” | Switzerland | 24 | NR | 79 | Cetuximab + capecitabine | NR | Allergic reaction/anaphylaxis |
| Lakomy, 2015^20^ | Open-label, prospective, observational, noninterventional study | NR “VECTIS” | Multi-country (Central and Eastern Europe) | 632 | 64.6% | 62.3 | Panitumumab | Systemic treatments after adverse event occurred (antibiotics, antihistamine, corticosteroids) | Allergic reaction/anaphylaxis |
| Lelli, 2008^21^ | Retrospective observational | NR | Italy | 144 | 61.8% | 63 | Cetuximab + irinotecan | NR | Allergic reaction/anaphylaxis |
| Lenz, 2004^22^ | Phase II non-randomized trial | NR | NR but likely multi-country (USA and Belgium) | 350 | 55.0% | 59 | Cetuximab | NR | Allergic reaction/anaphylaxis |
| Lenz, 2006^23^ | Phase II Trial and Translational Study | NCT00044863 | NR but likely Multi-country (United States and Belgium) | 346 | 53.5% | 59 | Cetuximab | Antihistamine | Hypersensitivity |
| Miguel, 2011^24^ | Retrospective chart review | NR | Portugal | 55 | NR | NR | Cetuximab | Antihistamine and corticosteroids | Hypersensitivity |
| Muro, 2009^25^ | Open-label, single-arm Phase II study | NR | Japan | 52 | 65.0% | 59 | Panitumumab | NR | Infusion reaction |
| Nakamura, 2017^26^ | Multicenter, open-label, observation-enriched randomized controlled design - prospective phase II study | NR “G13D-study” | Japan | 10 | 80.0% | 69 | Cetuximab | NR | Infusion-related reaction |
|  |  |  |  | 19 | 68.4% | 69 | Cetuximab + Irinotecan |  |  |
| Ocvirk, 2010^27^ | Phase II trial | NR “CECOG” | Multi-country (Central Europe) | 77 | 56.0% | 62 | Cetuximab + FOLFOX6 | NR | Infusion-related reaction; Allergic reaction/anaphylaxis |
|  |  |  |  | 74 | 61.0% | 62.5 | Cetuximab + FOLFIRI |  |  |
| O'Neil, 2007^28^ | Retrospective, multi-center study using pharmacy database and clinical trial records. | NR | United States | 69 | NR | NR | Cetuximab | Antihistamine alone or antihistamine + corticosteroids | Hypersensitivity |
| Pacek, 2014^29^ | Retrospective chart review | NR | Poland | 46 | 74.0% | 55.4 | Cetuximab +/- combination therapy with chemotherapy based on irinotecan, oxaliplatin, or capecitabine | NR | Infusion reaction |
| Peeters, 2010^30^ | Open-label, randomized, multi-center, phase III trial | NR | Multi-country | 541 | 60.9% | 60.5 | Panitumumab +  FOLFIRI | NR | Infusion-related reaction |
| Power, 2010^31^ | Retrospective observational | NR | United States | 35 | 76.5% | 59.5 | Panitumumab with prior cetuximab or cetuximab naive | Antihistamine and corticosteroid in patients with prior cetuximab | Infusion reaction |
|  |  |  |  | 22 | 76.5% | 59.5 | Cetuximab prior to panitumumab |  |  |
| Price, 2014^32^ | Randomized, multicenter, open-label, non-inferiority phase 3 study | NCT01001377 “ASPECCT” | Multi-country | 499 | 63.0% | 61 | Panitumumab | Cetuximab: antihistamine Panitumumab: not required | Infusion reaction |
|  |  |  |  | 500 | 64.0% | 60.5 | Cetuximab |  |  |
| Racca, 2008^33^ | Open-label, uncontrolled phase II trial | NR | Italy | 34 | 61.8% | 62.5 | Cetuximab | Antihistamine | Allergic reaction/anaphylaxis |
| Saltz, 2004^34^ | Phase II open-label trial | NR | United States | 57 | 61.0% | 56 | Cetuximab | Antihistamine | Allergic reaction/anaphylaxis |
| Saltz, 2007^35^ | Randomized phase II trial | NR “BOND-2” | United States | 43 | 60.0% | 64 | Cetuximab, bevacizumab + irinotecan | NR | Allergic reaction/anaphylaxis |
|  |  |  |  | 40 | 65.0% | 56 | Cetuximab + bevacizumab |  |  |
| Sastre, 2009^36^ | Phase II study | NR | Spain | 41 | 58.5% | 76 | Cetuximab | Antihistamine | Hypersensitivity |
| Smith, 2012^37^ | Prospective observational | NR “EREBUS” | France | 77 | 67.3% | 64 | Cetuximab + oxaliplatin | NR | Infusion reaction |
|  |  |  |  | 114 | 67.3% | 64 | Cetuximab + irinotecan |  |  |
| Sobrero, 2008^38^ | Open-label, phase III randomized controlled trial | NR “EPIC” | Multi-country | 648 | 62.5% | 61 | Cetuximab + Irinotecan | Antihistamine | Infusion reaction |
| Souglakos, 2007^39^ | Prospective phase II trial | NR | Greece | 40 | 57.5% | 65 | Cetuximab + oxaliplatin + capecitabine | Antihistamine; 5-hydroxytryptamine-3-receptor antagonist | Allergic reaction/anaphylaxis |
| Taira, 2014^40^ | Phase II study | UMIN000003819 “OGSG1001” | Japan | 31 | NR | 64 | Panitumumab + irinotecan | NR | Infusion-related reaction |
| Tang, 2017^41^ | Phase II trial | NR | China | 62 | 64.5% | 54.6 | Cetuximab | Antihistamine + corticosteroids | Infusion reaction |
| Tol, 2009^42^ | Open-label, phase III Randomized controlled trial | NCT00208546 “CAIRO2” | Netherlands | 368 | 63.3% | 62 | Cetuximab + Capecitabine + Oxaliplatin + Bevacizumab | NR | Hypersensitivity |
| Tsuji, 2016^43^ | Phase II study | UMIN000004197 “JACCRO CC-05” | Japan | 54 | 64.8% | 60 | Cetuximab + modified-FOLFOX6 | Antihistamine + corticosteroids | Infusion-related reaction |
| Van Cutsem, 2007^44^ | Open label Phase III multicenter randomized controlled trial | NR | Multi-country | 463 | 63.5% | 62 | Panitumumab + BSC | Premedication was not required | Infusion reaction |
| Van Cutsem, 2008^45^ | Open-label, single-arm Phase III extension study | NR | Multi-country | 176 | 63.0% | 62 | Panitumumab + BSC | Premedication in one patient after hypersensitivity occurred (unspecified) | Infusion reaction; hypersensitivity |
| Wierzbicki, 2011^46^ | Phase II study | NR | United States, Canada | 85 | 69.4% | 65.4 | Cetuximab | NR | Infusion-related reaction, hypersensitivity, allergic reaction/anaphylaxis |
| Wilke, 2008^47^ | Case series ("uncontrolled, multicenter study") | NR “MABEL” | Multi-country (Europe) | 1147 | 64.3% | 62 | Cetuximab + irinotecan | Antihistamine +/- corticosteroid | Infusion-related reaction |
| Yamaguchi, 2014^48^ | Prospective observational | NR | Japan | 2006 | 62.5% | 64 | Cetuximab either alone or in combination with irinotecan or FOLFIRI | Antihistamine + corticosteroid;  Antihistamine or  Corticosteroid alone | Infusion reaction |

*5-FU: fluorouracil; BSC: Best Supportive Care; FOLFIRI: folinic acid (leucovorin), fluorouracil, irinotecan; FOLFOX: folinic acid (leucovorin), fluorouracil, oxaliplatin; IFL: irinotecan, leucovorin, fluorouracil (dosage differs from FOLFIRI); NR: Not reported; UFOX: oxaliplatin, leucovorin, UFT (tegafur + uracil); XELIRI: irinotecan, capecitabine; XELOX: capecitabine, oxaliplatin.

References

1. Akiyoshi K, Hamaguchi T, Nagai Y, et al. A PROSPECTIVE MULTICENTER FEASIBILITY STUDY WITH SHORT-TIME INFUSION OF PANITUMUMAB (SHIP TRIAL). ANNALS OF ONCOLOGY: OXFORD UNIV PRESS GREAT CLARENDON ST, OXFORD OX2 6DP, ENGLAND, 2012:203-204.

2. Bachet JB, Afchain P, Fermanian C, et al. Cetuximab efficacy in patients treated routinely in university hospitals. Gastroenterol Clin Biol. 2007;31: 941-949.

3. Berlin J, Posey J, Tchekmedyian S, et al. Panitumumab with irinotecan/leucovorin/5-fluorouracil for first-line treatment of metastatic colorectal cancer. Clin Colorectal Cancer. 2007;6: 427-432.

4. Boccia RV, Cosgriff TM, Headley DL, Badarinath S, Dakhil SR. A phase II trial of FOLFOX6 and cetuximab in the first-line treatment of patients with metastatic colorectal cancer. Clin Colorectal Cancer. 2010;9: 102-107.

5. Bokemeyer C, Bondarenko I, Hartmann JT, et al. Efficacy according to biomarker status of cetuximab plus FOLFOX-4 as first-line treatment for metastatic colorectal cancer: the OPUS study. Ann Oncol. 2011;22: 1535-1546.

6. Cartwright T, Kuefler P, Cohn A, et al. Results of a phase II trial of cetuximab plus capecitabine/irinotecan as first-line therapy for patients with advanced and/or metastatic colorectal cancer. Clin Colorectal Cancer. 2008;7: 390-397.

7. Cohn AL, Shumaker GC, Khandelwal P, et al. An open-label, single-arm, phase 2 trial of panitumumab plus FOLFIRI as second-line therapy in patients with metastatic colorectal cancer. Clin Colorectal Cancer. 2011;10: 171-177.

8. Cunningham D, Humblet Y, Siena S, et al. Cetuximab monotherapy and cetuximab plus irinotecan in irinotecan-refractory metastatic colorectal cancer. N Engl J Med. 2004;351: 337-345.

9. Douillard JY, Siena S, Cassidy J, et al. Randomized, phase III trial of panitumumab with infusional fluorouracil, leucovorin, and oxaliplatin (FOLFOX4) versus FOLFOX4 alone as first-line treatment in patients with previously untreated metastatic colorectal cancer: the PRIME study. J Clin Oncol. 2010;28: 4697-4705.

10. Douillard JY, Zemelka T, Fountzilas G, et al. FOLFOX4 with cetuximab vs. UFOX with cetuximab as first-line therapy in metastatic colorectal cancer: The randomized phase II FUTURE study. Clin Colorectal Cancer. 2014;13: 14-26.e11.

11. Emons M, Dean B, Yu H, Barber B, Malin J, Zhao Z. PCN9 Adverse Events Among Patients with Metastatic Colorectal Cancer Treated with Monoclonal antibodies in Clinical Practice. Value in Health. 2011;14: A435.

12. Folprecht G, Lutz MP, Schoffski P, et al. Cetuximab and irinotecan/5-fluorouracil/folinic acid is a safe combination for the first-line treatment of patients with epidermal growth factor receptor expressing metastatic colorectal carcinoma. Ann Oncol. 2006;17: 450-456.

13. Galal KM. Inherent Resistance to Epidermal Growth Factor Receptor. J. Med. Sci. 2009;9: 165-174.

14. Gumus M, Geredeli C, Ucar M, et al. EGFR-targeted treatments in patients with metastatic colorectal cancer: Experience of panitumumab: American Society of Clinical Oncology, 2017.

15. Hazama S, Maeda H, Iwamoto S, et al. A Phase II Study of XELOX and Cetuximab as First-Line Therapy in Patients With KRAS Wild Type Metastatic Colorectal Cancer (FLEET2 Study). Clin Colorectal Cancer. 2016;15: 329-336.

16. Hecht JR, Patnaik A, Berlin J, et al. Panitumumab monotherapy in patients with previously treated metastatic colorectal cancer. Cancer. 2007;110: 980-988.

17. Jehn CF, Boning L, Kroning H, Possinger K, Luftner D. Cetuximab-based therapy in elderly comorbid patients with metastatic colorectal cancer. Br J Cancer. 2012;106: 274-278.

18. Jonker DJ, O'Callaghan CJ, Karapetis CS, et al. Cetuximab for the treatment of colorectal cancer. N Engl J Med. 2007;357: 2040-2048.

19. Kienle D, Winterhalder R, Koeberle D, et al. 1322 Cetuximab monotherapy and cetuximab plus capecitabine as first-line treatment in elderly patients with RAS-and BRAF wild-type metastatic colorectal cancer. Results of the multicenter phase II trial SAKK 41/10. European Journal of Cancer. 2015;51: S194-S195.

20. Lakomy R, Rogowski W, Piko B, Mihaylova Z, Pritzova E, Kvocekova L. Prospective noninterventional study on the use of panitumumab monotherapy in patients with recurrent or progressive colorectal cancer: the VECTIS study. Cancer Manag Res. 2015;7: 311-318.

21. Lelli G, Cataldo S, Carandina I, et al. The role of cetuximab in pre-treated refractory patients with metastatic colorectal cancer: outcome study in clinical practice. J Chemother. 2008;20: 374-379.

22. Lenz H, Mayer R, Gold P, et al. Activity of cetuximab in patients with colorectal cancer refractory to both irinotecan and oxaliplatin. Journal of Clinical Oncology. 2004;22: 3510-3510.

23. Lenz HJ, Van Cutsem E, Khambata-Ford S, et al. Multicenter phase II and translational study of cetuximab in metastatic colorectal carcinoma refractory to irinotecan, oxaliplatin, and fluoropyrimidines. J Clin Oncol. 2006;24: 4914-4921.

24. Miguel I, Carneiro M, Bento S. CETUXIMAB INFUSION RELATED REACTIONS, EXPERIENCE OF A SINGLE PORTUGUESE INSTITUTION. ANNALS OF ONCOLOGY: OXFORD UNIV PRESS GREAT CLARENDON ST, OXFORD OX2 6DP, ENGLAND, 2011:v125-v126.

25. Muro K, Yoshino T, Doi T, et al. A phase 2 clinical trial of panitumumab monotherapy in Japanese patients with metastatic colorectal cancer. Jpn J Clin Oncol. 2009;39: 321-326.

26. Nakamura M, Aoyama T, Ishibashi K, et al. Randomized phase II study of cetuximab versus irinotecan and cetuximab in patients with chemo-refractory KRAS codon G13D metastatic colorectal cancer (G13D-study). Cancer Chemother Pharmacol. 2017;79: 29-36.

27. Ocvirk J, Brodowicz T, Wrba F, et al. Cetuximab plus FOLFOX6 or FOLFIRI in metastatic colorectal cancer: CECOG trial. World J Gastroenterol. 2010;16: 3133-3143.

28. O'Neil BH, Allen R, Spigel DR, et al. High incidence of cetuximab-related infusion reactions in Tennessee and North Carolina and the association with atopic history. J Clin Oncol. 2007;25: 3644-3648.

29. Pacek A, Koziol M, Puskulluoglu M, et al. Assessment of skin-related toxicity in patients with metastatic colorectal cancer treated with cetuximab. Acta Dermatovenerol Croat. 2014;22: 137-144.

30. Peeters M, Price TJ, Cervantes A, et al. Randomized phase III study of panitumumab with fluorouracil, leucovorin, and irinotecan (FOLFIRI) compared with FOLFIRI alone as second-line treatment in patients with metastatic colorectal cancer. J Clin Oncol. 2010;28: 4706-4713.

31. Power DG, Shah MA, Asmis TR, Garcia JJ, Kemeny NE. Safety and efficacy of panitumumab following cetuximab: retrospective review of the Memorial Sloan-Kettering experience. Invest New Drugs. 2010;28: 353-360.

32. Price TJ, Peeters M, Kim TW, et al. Panitumumab versus cetuximab in patients with chemotherapy-refractory wild-type KRAS exon 2 metastatic colorectal cancer (ASPECCT): a randomised, multicentre, open-label, non-inferiority phase 3 study. Lancet Oncol. 2014;15: 569-579.

33. Racca P, Fanchini L, Caliendo V, et al. Efficacy and skin toxicity management with cetuximab in metastatic colorectal cancer: outcomes from an oncologic/dermatologic cooperation. Clin Colorectal Cancer. 2008;7: 48-54.

34. Saltz LB, Meropol NJ, Loehrer PJ, Sr., Needle MN, Kopit J, Mayer RJ. Phase II trial of cetuximab in patients with refractory colorectal cancer that expresses the epidermal growth factor receptor. J Clin Oncol. 2004;22: 1201-1208.

35. Saltz LB, Lenz HJ, Kindler HL, et al. Randomized phase II trial of cetuximab, bevacizumab, and irinotecan compared with cetuximab and bevacizumab alone in irinotecan-refractory colorectal cancer: the BOND-2 study. J Clin Oncol. 2007;25: 4557-4561.

36. Sastre J, Aranda E, Gravalos C, et al. First-line single-agent cetuximab in elderly patients with metastatic colorectal cancer. A phase II clinical and molecular study of the Spanish group for digestive tumor therapy (TTD). Crit Rev Oncol Hematol. 2011;77: 78-84.

37. Smith D, Rouyer M, Mitry E, et al. Use of cetuximab (CTX) in first-line therapy of metastatic colorectal cancer (mCRC): Patient characteristics, safety, and effectiveness in the EREBUS cohort: American Society of Clinical Oncology, 2012.

38. Sobrero AF, Maurel J, Fehrenbacher L, et al. EPIC: phase III trial of cetuximab plus irinotecan after fluoropyrimidine and oxaliplatin failure in patients with metastatic colorectal cancer. J Clin Oncol. 2008;26: 2311-2319.

39. Souglakos J, Kalykaki A, Vamvakas L, et al. Phase II trial of capecitabine and oxaliplatin (CAPOX) plus cetuximab in patients with metastatic colorectal cancer who progressed after oxaliplatin-based chemotherapy. Ann Oncol. 2007;18: 305-310.

40. Taira K, Yoshida M, Sugimoto N, et al. A phase II study of panitumumab plus irinotecan for metastatic colorectal cancer with wild KRAS, resistant to fluoropyrimidine, oxaliplatin, and irinotecan in Japanese (OGSG1001): American Society of Clinical Oncology, 2014.

41. Tang XM, Chen H, Liu Y, et al. The cardiotoxicity of cetuximab as single therapy in Chinese chemotherapy-refractory metastatic colorectal cancer patients. Medicine (Baltimore). 2017;96: e5946.

42. Tol J, Koopman M, Cats A, et al. Chemotherapy, bevacizumab, and cetuximab in metastatic colorectal cancer. N Engl J Med. 2009;360: 563-572.

43. Tsuji A, Sunakawa Y, Ichikawa W, et al. Early Tumor Shrinkage and Depth of Response as Predictors of Favorable Treatment Outcomes in Patients with Metastatic Colorectal Cancer Treated with FOLFOX Plus Cetuximab (JACCRO CC-05). Target Oncol. 2016;11: 799-806.

44. Van Cutsem E, Peeters M, Siena S, et al. Open-label phase III trial of panitumumab plus best supportive care compared with best supportive care alone in patients with chemotherapy-refractory metastatic colorectal cancer. J Clin Oncol. 2007;25: 1658-1664.

45. Van Cutsem E, Siena S, Humblet Y, et al. An open-label, single-arm study assessing safety and efficacy of panitumumab in patients with metastatic colorectal cancer refractory to standard chemotherapy. Ann Oncol. 2008;19: 92-98.

46. Wierzbicki R, Jonker DJ, Moore MJ, et al. A phase II, multicenter study of cetuximab monotherapy in patients with refractory, metastatic colorectal carcinoma with absent epidermal growth factor receptor immunostaining. Invest New Drugs. 2011;29: 167-174.

47. Wilke H, Glynne-Jones R, Thaler J, et al. Cetuximab plus irinotecan in heavily pretreated metastatic colorectal cancer progressing on irinotecan: MABEL Study. J Clin Oncol. 2008;26: 5335-5343.

48. Yamaguchi K, Watanabe T, Satoh T, et al. Severe infusion reactions to cetuximab occur within 1 h in patients with metastatic colorectal cancer: results of a nationwide, multicenter, prospective registry study of 2126 patients in Japan. Jpn J Clin Oncol. 2014;44: 541-546.
